# Closing the gap between rocks and clocks using total-evidence dating

**DOI:** 10.1098/rstb.2015.0136

**Published:** 2016-07-19

**Authors:** Fredrik Ronquist, Nicolas Lartillot, Matthew J. Phillips

**Affiliations:** 1 Department of Bioinformatics and Genetics, Swedish Museum of Natural History, PO Box 50007, 104 05 Stockholm, Sweden; 2 Laboratoire de Biométrie et Biologie Evolutive, UMR CNRS 5558, Université Claude Bernard Lyon 1, F-69622 Villeurbanne Cedex, France; 3 School of Earth, Environmental and Biological Sciences, Queensland University of Technology, 2 George Street, Brisbane, Queensland 4000, Australia

**Keywords:** total-evidence dating, mammals, fossilized birth–death, deep root attraction

## Abstract

Total-evidence dating (TED) allows evolutionary biologists to incorporate a wide range of dating information into a unified statistical analysis. One might expect this to improve the agreement between rocks and clocks but this is not necessarily the case. We explore the reasons for such discordance using a mammalian dataset with rich molecular, morphological and fossil information. There is strong conflict in this dataset between morphology and molecules under standard stochastic models. This causes TED to push divergence events back in time when using inadequate models or vague priors, a phenomenon we term ‘deep root attraction’ (DRA). We identify several causes of DRA. Failure to account for diversified sampling results in dramatic DRA, but this can be addressed using existing techniques. Inadequate morphological models also appear to be a major contributor to DRA. The major reason seems to be that current models do not account for dependencies among morphological characters, causing distorted topology and branch length estimates. This is particularly problematic for huge morphological datasets, which may contain large numbers of correlated characters. Finally, diversification and fossil sampling priors that do not incorporate all the available background information can contribute to DRA, but these priors can also be used to compensate for DRA. Specifically, we show that DRA in the mammalian dataset can be addressed by introducing a modest extra penalty for ghost lineages that are unobserved in the fossil record, for instance by assuming rapid diversification, rare extinction or high fossil sampling rate; any of these assumptions produces highly congruent divergence time estimates with a minimal gap between rocks and clocks. Under these conditions, fossils have a stabilizing influence on divergence time estimates and significantly increase the precision of those estimates, which are generally close to the dates suggested by palaeontologists.

This article is part of the themed issue ‘Dating species divergences using rocks and clocks’.

## 1. Introduction

Until recently, dating phylogenies with fossils has been based on the notion of associating particular calibration nodes in molecular-clock trees with age estimates derived from the fossil record. Nowadays, such ‘node dating’ is usually done within a Bayesian framework, using relaxed clock models and sophisticated prior probability distributions describing the information in the fossil record about the age of the calibration nodes. Node dating involves a stepwise analysis of divergence times: initially, calibration priors are derived from the fossil record and associated with particular tree nodes, and then these priors are combined with molecular data to produce posterior distributions on dated trees.

Expressing the dating information in the fossil record as probability distributions on node ages is quite challenging [1]. Each calibration distribution should summarize the information from multiple fossils, while accommodating the uncertainty in the dating of each fossil, in its phylogenetic placement, and in the length of any side branches connecting it to the tree of extant taxa. Ideally, the calibration prior should also factor in the spatial and temporal sampling of fossils, and the implications of the fossil record with respect to likely diversification processes in the past.

In recent years, evolutionary biologists have started exploring an alternative approach to dating phylogenies with fossils that we will refer to as ‘total-evidence dating’ (TED) [2–4]. TED is often called ‘tip dating’, even though TED or ‘integrative dating’ are more descriptive. TED involves simultaneous analysis of fossil and recent taxa, allowing evolutionary biologists to incorporate a wide range of sources of dating information into a unified statistical analysis.

TED requires explicit coding of the character evidence that informs fossil affinities, which is typically hard work. However, the payoff is considerable. TED involves direct analysis of the available evidence; there is no need for secondarily derived calibration nodes, although it is of course possible to combine TED with node dating. TED integrates over the topological uncertainty in the placement of fossils, and the length of any side branches connecting them to the extant tree. This allows fossils with more certain placements and with traits that are closer to ancestral forms to exert more influence over the dating than fossils with uncertain affinities and many unique features. TED allows one to explicitly represent the uncertainty concerning the age of each individual fossil and, using fossilized birth–death (FBD) models, TED can also incorporate information about speciation, extinction and sampling processes [5].

In one of the first TED analyses, we showed that divergence time estimates from TED were more robust to variation in prior assumptions and more precise (and presumably more accurate) than estimates from node dating [4]. We also showed that apparently erroneous or questionable assumptions about fossil placements can seriously bias divergence time estimates in node dating. This supports the expectation that an integrative approach, like TED, should be less sensitive to potential methodological problems than a complex stepwise procedure like node dating, in which it is more difficult to capture uncertainty in the initial steps (phylogenetic inference, placement of fossils in the tree, derivation of calibration distributions), so that it can be carried over properly to the final analysis. Thus, TED would appear to have the potential to close the gap between rocks and clocks often observed in node dating studies.

Recent work has shown that this is not always the case in practice. In fact, TED is often reported to result in divergence time estimates that are more uncertain and more at odds with the fossil record than those from node dating or a combination of TED and node dating [6,7]. The aim of this paper is to examine why this might be the case.

For several reasons, we chose to focus on the radiation of mammals, more specifically on the radiation of the Eutheria (crown-group and stem-group placentals). There may be no other group with so much data available for a TED analysis, and so much controversy surrounding the divergence time estimates. Recently, an exceptional morphological (phenomic) dataset (the O'Leary *et al*. dataset) was published for fossil and recent mammals [8]. With 4541 characters in total, scored for 46 extant and 40 fossil taxa, this may be the largest phenomic dataset published to date: it includes 1284 cranial characters, 1451 dental characters, 925 postcranial characters and 881 soft characters (including behavioural and other characters). There are also rich genetic and genomic data available for extant placentals [9,10].

The debate concerning the age of crown placentals has a long history and is not settled yet. Some palaeontologists [8,11] argue for an origin adjacent to the Cretaceous–Palaeogene mass extinction (K–Pg event) around 66 Ma, followed by an explosive radiation, producing extant mammal orders during a time interval of a few hundred thousand years. This explosive radiation scenario is supported if one minimizes the length of any postulated lineages that are unobserved in the fossil record (‘ghost lineages’) [8]. In contrast, node dating based on genetic or genomic data place the placental origin at around 101 [9] or 90 Ma [10]; these trees postulate that a substantial portion of the interordinal placental diversification took place before the K–Pg event. The abundance of placental fossils in the Palaeogene and the absence of them in the Cretaceous provide one of the strongest pieces of evidence supporting the explosive radiation scenario ([8] but see [12]), whereas the high rates of molecular evolution around the K–Pg boundary required by such a scenario is one of the most powerful arguments against it ([13] but see [14]).

The O'Leary *et al*. dataset [8] has not been subjected yet to TED analysis. However, a TED analysis of the placental radiation based on a similar dataset was recently published [15]. The phenomic dataset in the latter study comprised 421 characters coded for 102 taxa, most of them fossils. The divergence time estimates obtained when these morphological data were analysed alone or in combination with molecular data under an unconstrained TED model were unrealistically old (around 164 Ma), suggesting that the placental radiation might be a suitable target for investigating why TED misbehaves in some cases.

Using a slightly modified version of the O'Leary *et al*. dataset, we show here that there is a tendency for TED divergence time estimates, under certain conditions, to be pushed back to very old strata and to be very imprecise. We term this phenomenon ‘deep root attraction’ (DRA). By exploring various prior models, we characterize the causes of DRA. DRA occurs when there are model errors or conflicts in the data that are difficult to reconcile, and ghost lineages carry little cost. A major driver of DRA appears to be inadequacies of current morphological models, a problem that is difficult if not impossible to address. However, by increasing the cost of ghost lineages, we show that it is possible to address DRA and obtain a stable TED solution that is robust to variation in prior assumptions. This solution places the radiation of crown placentals at around 85 Ma, which is intermediate between the explosive radiation scenario and the age estimates suggested by most previous node dating analyses (for exceptions, see [16,17]).

## 2. Methods

### (a) Data

The dataset of O'Leary *et al*. [8] includes 46 recent taxa and 40 fossil taxa. We were worried that it would be difficult to model the radiation of mammals into stem forms of monotremes, marsupials and placentals because these early events involve few lineages and long time periods according to both molecular and palaeontological studies. Therefore, we removed all extant and fossil taxa outside of the Eutheria (placentals plus stem-group placentals). The fossil *Eomaia scansoria* was placed outside of Theria (placentals + marsupials) in the analysis of O'Leary *et al*. [8] but it has previously been considered a member of Eutheria or Placentalia in analyses that densely sample stem therians [18,19], and we therefore included it in our dataset. To improve the fit between the data and our model of diversified sampling, we removed a very recent split among extant taxa by deleting *Galeopterus variegatus* from the dataset. Similarly, we omitted the very young (16.4 Ma) fossil *Hapalops elongatus*. The diversified sampling model [20] assumes that all deep splits in the tree have been sampled. The actual age of the most recent split in the tree, beyond which no more lineages are sampled, is inferred from data. Spurious young speciation events can seriously distort the inference of BD parameters under this model because they erroneously extend the period during which the process is assumed to be completely sampled. The resulting dataset included 74 eutherian or potentially eutherian taxa, 33 fossils and 41 extant taxa. The character data comprised 4541 discrete morphological characters and 36 860 DNA sites from 27 nuclear genes, including 22 protein-coding genes and five untranslated regions (UTRs) [8].

### (b) Substitution models

The morphological data were modelled using the Mk model [21], correcting for the coding bias resulting from only variable characters being present in the matrix. The matrix actually did include some constant characters, but they were removed prior to analysis. Rate variation across morphological characters was modelled using a discrete gamma model with four categories.

The 22 protein-coding genes were divided into three partitions corresponding to different codon positions, with the five UTRs treated in a separate partition. Each of the four molecular data partitions was associated with a unique nucleotide substitution model. Stationary state frequencies were allowed to differ, and we sampled across all different ways of partitioning the exchangeability rates [22]. Rate variation across sites was modelled using a gamma model (four discrete rate categories) with a proportion of invariable sites. Each of the four molecular data partitions was associated with a unique set of exchangeability rates, stationary state frequencies, gamma shape and proportion of invariable sites. The base rate of each partition was drawn from a Dirichlet distribution, under the constraint that the average rate across characters was 1.0.

### (c) Non-clock and clock models

For non-clock analyses, we assumed equal prior probability for all topologies. In all strict and relaxed clock analyses, we used a diffuse offset exponential prior on the age of the root of the tree, with a mean of 164 Ma and an offset of 64 Ma based on the oldest generally accepted crown placentals [8]. When fossils were included in the analyses, the minimum age of the induced prior was constrained by the age of the oldest fossil, *Eomaia scansoria*, which is dated to 122 Ma. The clock rate was associated with a lognormal prior with a mean of −6.0 and a standard deviation of 0.5 on the natural logarithm scale. This prior has an expectation of 0.25% substitutions per site per million years.

All relaxed clock analyses assumed an independent gamma rates (white noise) model [23] with the variance increase parameter having an exponential prior with rate 10 (expectation 0.1). Because of rooting problems (see Results), we constrained all relaxed clock analyses to have a monophyletic Laurasiatheria + Euarchontoglires in the tree of extant taxa, unless noted otherwise. The positions of fossil taxa were never constrained.

### (d) Dating priors

For dating, we used a uniform tree prior [4], a BD prior and an FBD prior [24]. The uniform tree prior does not have any parameters. For the naive BD and FBD priors, we used an exponential prior with rate 10 (expectation 0.1) for the net diversification rate, *d* (*d* = *λ* – *μ*, where *λ* is the birth rate and *μ* is the death rate), and a uniform beta prior, that is, a Beta(1,1) prior, for the turnover, *r* (*r* = *μ*/*λ*). For the fossil sampling probability, *f* (*f* = *ψ*/(*μ* + *ψ*), where *ψ* is the fossilization rate times the probability of subsequent discovery of the fossil), we also used a Beta(1,1) prior.

The ages of fossils were updated from O'Leary *et al.* [8] (see electronic supplementary material, appendix S1). We did not associate the age estimates with any uncertainty, assuming that this would be a negligible source of error in our analyses, and we take our results to confirm this assumption at least with respect to our main conclusions. However, the uncertainty of age estimates may clearly be important in many contexts [6].

### (e) Tip sampling assumptions

For both the BD and FBD priors, we explored three different assumptions concerning tip sampling, namely that the tips represent: (i) a complete sample, (ii) a random sample or (iii) a diversified sample of the entire tree [5,20]. Given that there are approximately 5000 described species of placentals, we considered the 41 extant taxa in our dataset to represent approximately 1% of the total diversity of the group in the randomized and diversified tip sampling scenarios.

### (f) Informative dating priors

To increase the penalty for ghost lineages, we explored four different variants of the basic BD and FBD models with vague priors. First, we increased the prior probability of a low extinction rate by using a Beta(1,100) prior instead of a flat prior for the turnover, *r*. Second, we increased the prior probability of a high fossilization rate by using a Beta(100,1) prior instead of a flat prior for the fossil sampling probability, *f*. Third, we combined these two assumptions in a low extinction rate/high fossil sampling rate prior. Finally, we explored the effects of assuming a rapid net diversification rate, *d*, by fixing it to 0.1 while using flat Beta(1,1) priors for *r* and *f*. All analyses were run both with fossils under the FBD model, and without fossils under the corresponding BD model.

### (g) Alternative models

We explored two alternative models that could potentially address DRA by providing more realistic assumptions about diversification processes or rate variation across the tree. We first allowed the diversification process to vary across time by using a skyline FBD model [25] with three intervals: older than 70, 70–55 and younger than 55 Ma. FBD parameters were estimated separately for each of these intervals, using the standard priors (Exp(10) for *d* and Beta(1,1) for *r* and *f*). Second, we decoupled the relaxed clocks for morphology and molecules, allowing morphological and molecular rates to have different trajectories across the tree.

### (h) Markov chain Monte Carlo sampling

All analyses were performed with pre-release versions of MrBayes v. 3.2.6 (svn revision 1067 and following) [26], the source code of which is available from the program website (http://mrbayes.net). The code is now part of the latest MrBayes release. To improve topological convergence when fossils are included, we added a couple of topology moves that specifically target fossil subtrees but that are, otherwise, identical to existing topology moves.

For each model, we ran four independent analyses, each using four Metropolis-coupled chains. We ran the chains for 10–30 M generations each and sampled them every 500 generations, discarding 25% of the samples as burn-in. When fossils were included in the analysis, tree samples were summarized both with fossils included and with fossils first pruned away from all tree samples. Data files and MrBayes run files for all analyses are provided in the electronic supplementary material.

All tree figures were drawn using FigTree [27]. Kernel densities of posterior distributions were estimated from the sampled values using the defaults of the ‘density’ function in R [28], with the default bandwidth adjusted by a factor of 2.0–5.0.

## 3. Results

### (a) Conflict between morphology and molecules

**Figure 1.**
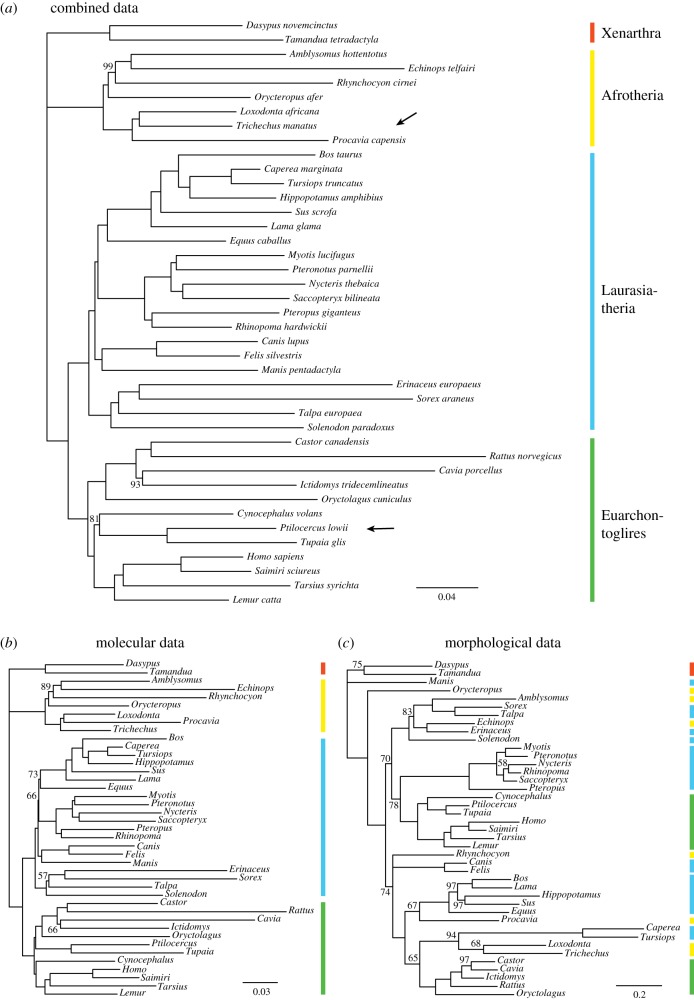
Phylogenetic signal in the dataset. (*a*) Non-clock analysis of extant taxa using combined data. Posterior probabilities (in %) indicated on branches if below 100%. (*b*) Ditto using molecular data only. (*c*) Ditto using morphological data only. Note that the morphological tree (*c*) has longer branches and is more poorly supported than the molecular tree (*b*), indicating weak and potentially misleading phylogenetic signal owing to morphological convergence. The morphological tree is also strongly in conflict with monophyly of the four placental superorders (indicated by coloured bars). Nevertheless, the morphological signal is strong enough to change a few details in the combined tree (*a*), namely the positions of Scandentia (*Ptilocercus* and *Tupaia*) and of the rock hyrax (*Procavia*; arrows). (Online version in colour.)

The non-clock analyses demonstrate strong conflict between the molecular and the morphological signal in the eutherian dataset (figure 1). Whereas the total-evidence (figure 1*a*) and molecular (figure 1*b*) trees both retrieve the four mammalian superorders that have been recognized in most recent analyses (Xenarthra, Afrotheria, Laurasiatheria and Euarchontoglires), the morphological tree (figure 1*c*) presents quite different groupings, often consistent with results from classical morphological cladistic analyses of placental relationships [29]. The morphological evidence sprinkles the afrotherian taxa across the tree, and even if we disregard the Afrotheria, the Euarchontoglires and Laurasiatheria remain intermixed. Among the Laurasiatheria, the pangolin (*Manis*) occupies a basal position in the tree together with Xenarthra (anteaters, sloths and armadillos). Furthermore, primates and their relatives (Euarchonta) group with bats (Chiroptera) rather than with Glires (rodents and lagomorphs), splitting the Euarchontoglires in two.

Many of the higher groupings in the morphology tree are poorly supported, with posterior probabilities well below 80% (figure 1*c*). The branches in the tree are also long (electronic supplementary material, figure S1*b*), suggesting that there is considerable plasticity in morphology, with a potential for misleading convergence. This contrasts with the molecular tree, which is well resolved and has a much smaller proportion of characters changing along each branch (figure 1*b* and electronic supplementary material, figure S1*a*). The branch length estimates are quite precise both for the morphological and molecular data (electronic supplementary material, figure S1*c*,*d*). Adding fossils to the morphology tree did not change the inferred relationships among extant taxa in any major way (electronic supplementary material, figure S2*a*).

The analysis based on combined data resulted in a tree that was largely the same as the molecular tree (figure 1*a*). However, despite the morphological characters being fewer (4.5 versus 36.9 k) and more homoplastic than the molecular characters, they were nevertheless able to change a few details in the molecular tree. Specifically, the combined tree supported the monophyly of Euarchonta (primates with tree shrews and flying lemurs), whereas the molecular tree grouped tree shrews with rodents and lagomorphs (arrows, figure 1*a*). The morphological data also changed the position of the rock hyrax (*Procavia*) slightly. Adding fossils to the combined-data analysis only resulted in one minor topological change in the extant tree, affecting the position of the aardvark (*Orycteropus*) within Afrotheria (electronic supplementary material, figure S2*b*).

### (b) Rooting clock trees

**Figure 2.**
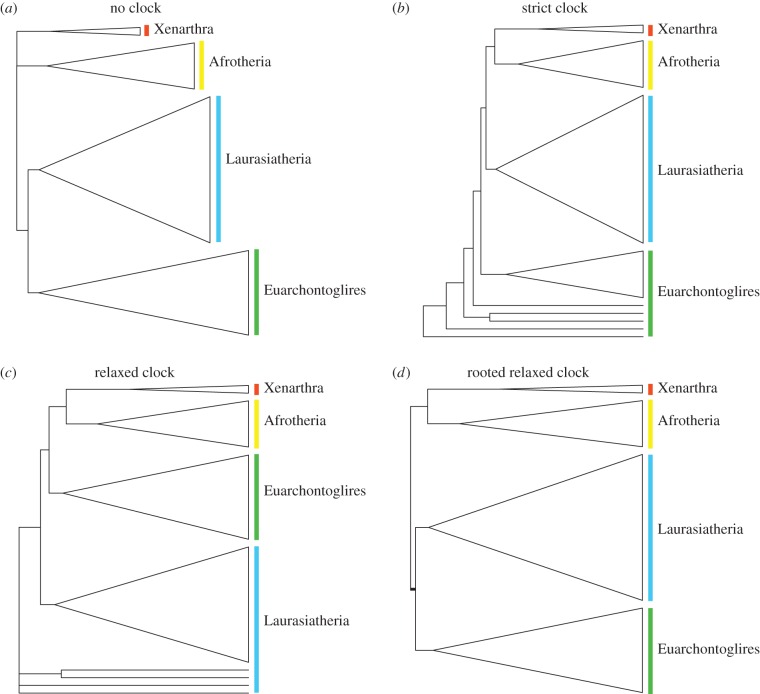
Rooting artefacts in clock trees. (*a*) Analysis of relationships among extant taxa under a non-clock model. (*b*) Ditto under a strict-clock model. (*c*) Ditto under a relaxed-clock model (independent gamma rates). (*d*) Ditto under a relaxed-clock model (independent gamma rates), enforcing the topological constraint that Boreoeutheria (Euarchontoglires + Laurasiatheria) are monophyletic. The non-clock tree (*a*) can be rooted by appropriate choice of outgroup, so that placental superorders (coloured bars) are monophyletic. Clock models produce rooted trees without the need for specifying an outgroup. However, both strict (*b*) and relaxed clocks (*c*) result in topological artefacts close to the root when no rooting constraints are enforced. This can be solved in the relaxed-clock analysis by adding a single topological constraint close to the root of the tree (*d*). (Online version in colour.)

Non-clock trees based on molecular data and combined data can readily be rooted, so that the four mammalian superorders are monophyletic (figure 2*a*). Unconstrained strict-clock and relaxed-clock analyses, however, produce rooted trees with various apparent topological artefacts owing to incorrect rooting (figure 2*b*,*c*). Depending on the exact model used, the clock trees may be rooted between Euarchontoglires and Laurasiatheria, or within one of these two superorders (often within Rodentia or Eulipotyphla (hedgehogs, shrews and moles)). The artefacts occur both in strict-clock trees and in relaxed-clock trees. Adding a single rooting constraint in the relaxed-clock analyses, forcing Boreoeutheria (Euarchontoglires + Laurasiatheria) to be monophyletic, is sufficient to stabilize the rooting and retrieve a tree topology of extant taxa that is fully congruent with the non-clock tree, and consistent with monophyly of all four mammalian superorders (figure 2*d*). Such a rooting constraint was used for extant taxa in all subsequent relaxed-clock analyses; fossil taxa were allowed to attach to any part of the extant tree.

### (c) Tip sampling effects

**Figure 3.**
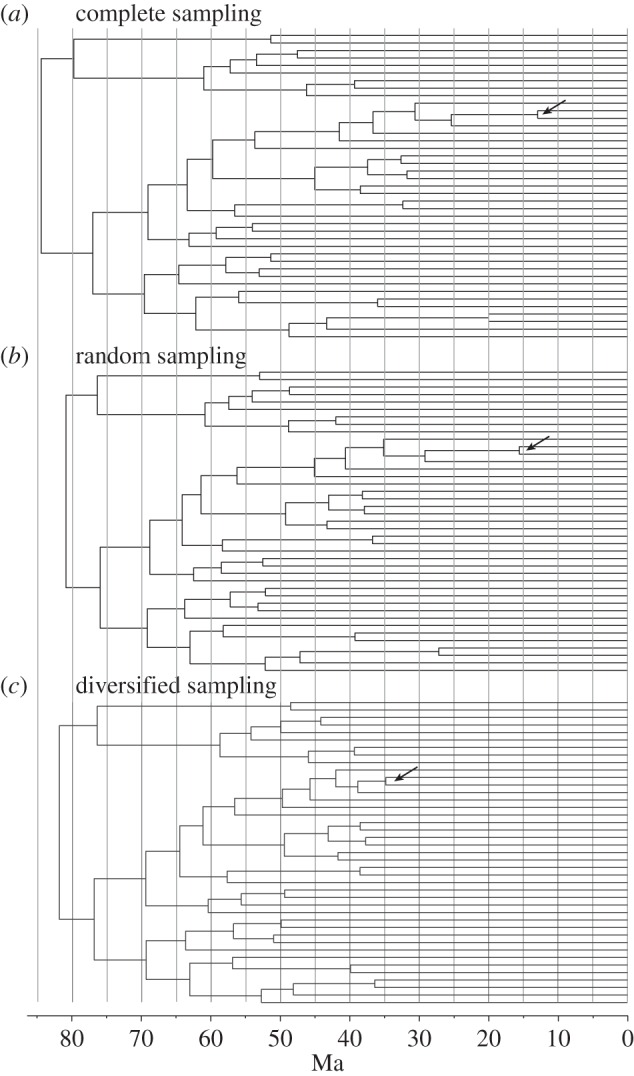
The effects of biases in tip sampling when dating under a birth–death model. (*a*) Relaxed-clock dating analysis of extant taxa assuming complete sampling of tips. (*b*) Ditto assuming random sampling of tips. (*c*) Ditto assuming diversified sampling of tips. In studies of higher taxa, it is standard practice to choose exemplars that span as much as possible of the phylogenetic diversity of the studied group. Such diversified sampling results in recent lineage splits being absent in the tree. If we analyse diversified data assuming that the tips represent a complete or a random sample, major dating errors may result. Specifically, there will be a tendency to spread splits evenly over time, so that deep divergences become too old and/or recent divergences too young. For instance, note that the most recent split (arrow)—between the whale (*Caperea marginata*) and the dolphin (*Tursiops truncatus*)—is dated to 35 Ma under diversified sampling (*c*), consistent with the fossil record [8], whereas it is dated to 12 Ma under complete sampling (*a*) and 16 Ma under random sampling (*b*).

The assumption concerning the tip sampling procedure has a major influence on the divergence time estimates obtained under BD models (figure 3). When fossils are not included in the analysis, the root date is not affected much for the studied dataset, but the estimates of the most recent divergence times differ substantially. The effect is most clearly seen in the estimated age of the split between the whale (*Caperea*) and the dolphin (*Tursiops*), the most recent of all speciation events (arrows in figure 3). If tips are assumed to represent a complete sample of lineages, then the split is estimated to (all posterior age distributions are summarized using the median, followed by the 95% region of highest posterior density) 13 (5, 26) Ma (figure 3*a*). If we account for the fact that the tips only represent approximately 1% of all known species, but still assume that they represent a random sample, the same split is estimated to 16 (5, 27) Ma (figure 3*b*). However, when we also account for the tips being chosen to maximize diversity, the estimate is pushed back to 35 (22, 60) Ma (figure 3*c*), which is more in line with expectations based on the fossil record [8].

### (d) Deep root attraction

**Figure 4.**
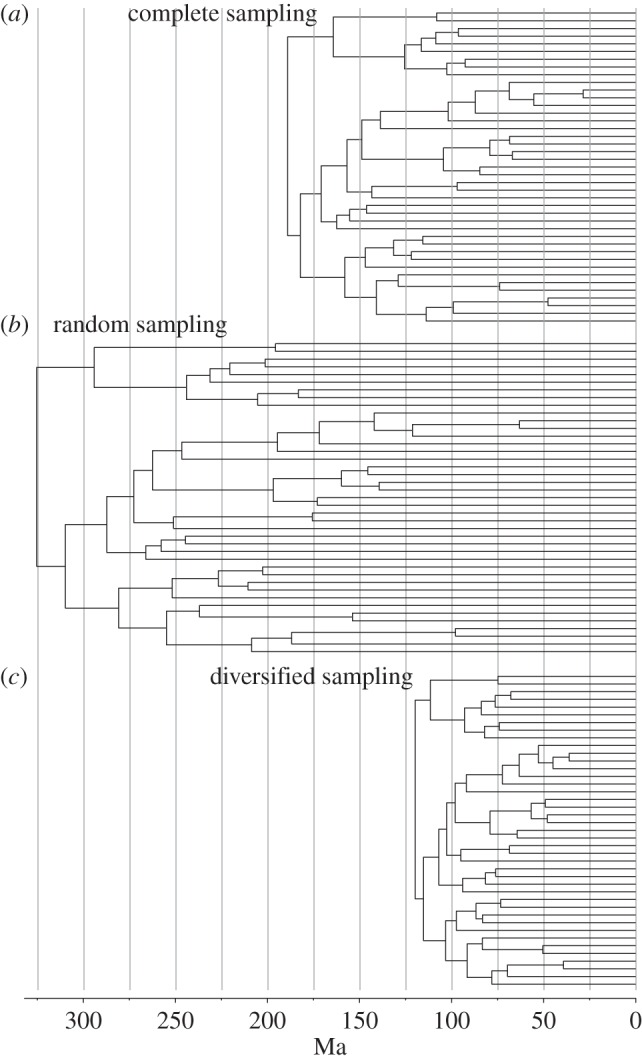
The effects of model inadequacies in total-evidence dating (TED). (*a*) TED under a birth–death model assuming complete sampling of tips (only extant tree shown). (*b*) Ditto assuming random sampling of tips. (*c*) Ditto assuming diversified sampling of tips. Under TED, there is a tendency for some model inadequacies to push divergence time estimates towards unreasonably old strata, a phenomenon we term ‘deep root attraction’. For the eutherian data, it appears that the major underlying cause of DRA is the difficulty of reconciling the conflict between molecular and morphological signals under standard models. The problem is exaggerated by inappropriate modelling of the tip sampling protocol (*a,b*) but it is presumably also influencing the results under a more realistic tip sampling model (*c*).

When fossils are added to the analysis under an FBD model with vague priors, the effect of changing the tip sampling assumption becomes dramatic (figure 4). Specifically, erroneous assumptions about the sampling procedure (figure 4*a*,*b*) push divergence time estimates back in time to strata that would be considered completely unrealistic by most workers. Even under the diversified sampling assumption, divergence time estimates appear old (figure 4*c*), especially for the deepest splits in the tree, and they are quite uncertain (table 1). For instance, the crown Placentalia are dated in this analysis to 118 (95, 178) Ma, which is older than the estimates resulting from many molecular-clock analyses and much older than the ages suggested by palaeontologists [8], but the credible interval is quite wide.

**Table 1.** Prior and posterior distributions for the ages of key clades under the uninformative and rapid diversification TED model priors. The induced joint prior distributions were estimated under the full model, with fossils included, using Markov chain Monte Carlo sampling with log-likelihood of characters set to 0.0. Defined in this way, the prior distribution contains a fair amount of dating information from FBD model parameter priors and from the number of sampled fossils and their ages. Estimated distributions are summarized by the median followed by the 95% region of highest posterior density.

clade	uninformative		rapid diversification	
	induced prior	posterior	induced prior	posterior
Placentalia	111 (86, 141)	118 (95, 178)	85 (66, 106)	85 (76, 93)
Xenarthra	46 (33, 84)	72 (44, 109)	39 (30, 63)	43 (35, 55)
Afrotheria	70 (44, 99)	86 (70, 120)	52 (37, 74)	66 (61, 74)
Afroinsectiphila	54 (37, 80)	79 (62, 112)	42 (33, 60)	56 (45, 66)
Paenungulata	50 (35, 77)	76 (63, 101)	41 (32, 59)	61 (47, 68)
Boreoeutheria	95 (73, 119)	113 (92, 174)	72 (58, 89)	81 (74, 90)
Laurasiatheria	86 (65, 107)	106 (85, 162)	64 (48, 79)	75 (68, 82)
Eulipotyphla	57 (38, 81)	91 (71, 156)	43 (34, 60)	56 (44, 69)
Euungulata	65 (43, 88)	94 (74, 134)	47 (37, 64)	65 (59, 72)
Cetartiodactyla	56 (39, 77)	74 (57, 100)	43 (34, 56)	54 (47, 60)
Chiroptera	62 (42, 84)	75 (55, 106)	46 (36, 63)	55 (43, 65)
Ferae	52 (35, 76)	94 (74, 137)	41 (32, 57)	68 (61, 75)
Carnivora	39 (32, 59)	71 (38, 102)	36 (30, 45)	44 (35, 61)
Euarchontoglires	82 (60, 105)	104 (83, 146)	61 (42, 76)	76 (69, 85)
Glires	65 (41, 89)	98 (79, 130)	47 (35, 65)	73 (66, 81)
Rodentia	52 (36, 74)	89 (70, 116)	41 (32, 55)	67 (61, 73)
Euarchonta	69 (46, 91)	91 (70, 128)	50 (37, 67)	65 (58, 73)
Primates	50 (36, 72)	77 (59, 109)	40 (32, 54)	58 (52, 65)

The same pull towards unrealistic deep-root scenarios occurs under the uniform clock tree prior, which must also be described as vague or uninformative about divergence times. Without fossils, the estimated crown age of Placentalia under this prior is 84 (64, 151) Ma, which is not unreasonable. However, inclusion of fossils in the analysis, without changing any of the prior settings, moves the estimate back to 284 (203, 374) Ma, which would imply more than 200 Myr of evolution of crown placentals without leaving a trace in the fossil record.

We term the pull towards deep-root scenarios that we observe under TED with vague or erroneous priors DRA. To explore the causes of DRA in the study dataset, we examined four informative diversification models that put less prior probability on deep-root scenarios with long ghost lineages: a model with a low expected extinction rate, a model with a high expected fossil sampling rate, a model combining both of these expectations and a model assuming a high net diversification rate (up to the time of the last speciation event observed in the diversified tree; after that event, the tree is no longer informative about diversification rates because of the absence of speciation events).

**Figure 5.**
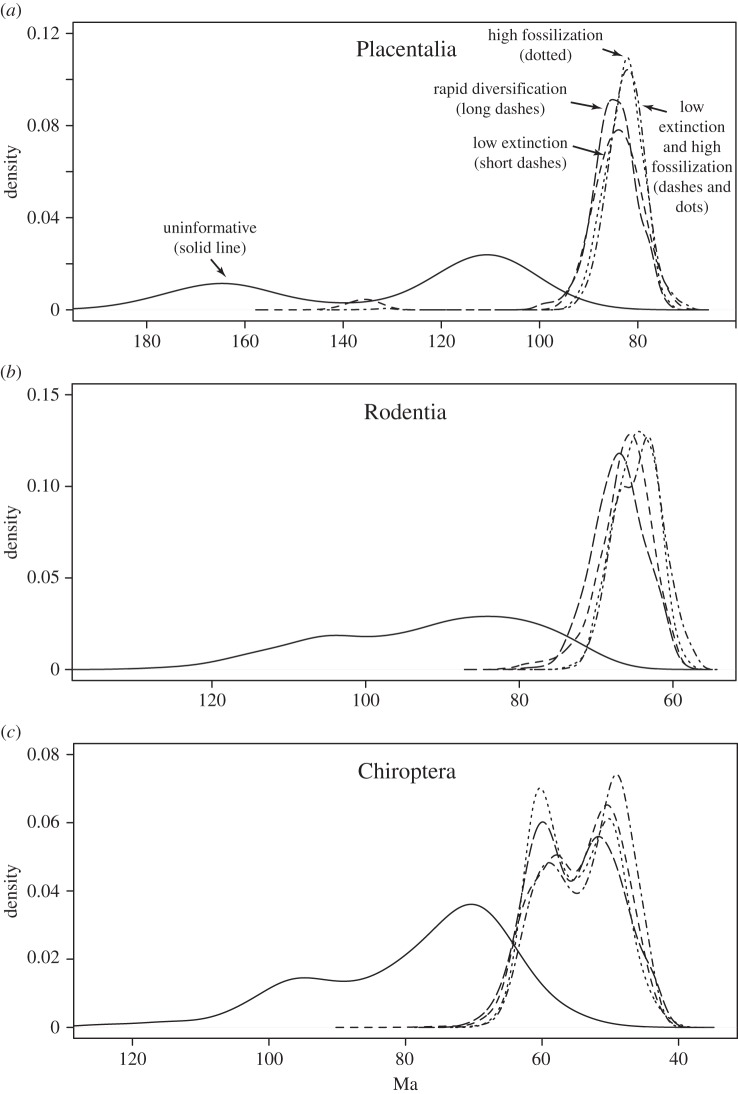
The influence of diversification and fossil sampling parameter priors. (*a*) Divergence time estimates (posterior densities) for crown Placentalia under different priors on diversification and fossil sampling parameters. (*b*) Ditto for crown Rodentia. (*c*) Ditto for crown Chiroptera. Ideally, with a large tree and a rich fossil sample, there will be enough information to infer diversification and fossil sampling parameters under an uninformative prior, but the signal in the eutherian data is not strong enough to resist DRA under such conditions. Instead, an uninformative prior results in a poorly defined, bimodal deep-root scenario in the posterior (solid line). It is sufficient to introduce a moderate extra penalty for unobserved ghost lineages to shift the posterior to a well-defined solution with a minimal gap between rocks and clocks. Such penalties may involve assumptions of low extinction rates, high fossil sampling probabilities, high net diversification or any combination of these (various dashed lines); the result is similar under all these scenarios. Bimodal distributions correspond to cases where there are alternative likely placements for key fossils.

Detailed analysis of divergence time estimates for three key clades under these models (figure 5) shows that the vague prior is associated with a posterior that is both diffuse and bimodal. The multiple peaks in the distribution are caused by different topological placements of key fossils. For instance, the age estimate for crown placentals (figure 5*a*) is affected by the position of *Eomaia*, which can be placed either within or outside of Placentalia, and both solutions have significant posterior probability.

In contrast, the models with informative priors all give posterior distributions that have rather small variance and that are often unimodal (figure 5). When they are bimodal, the cause is the same as for the model with vague priors. For instance, the bimodal posterior for the age of bats (figure 5*c*) is due to the uncertainty in placing the two bat fossils: either inside or outside crown bats.

Importantly, although the informative-prior models are quite different among themselves, they produce similar posterior distributions. This is true not only for the key clades examined here, but also for the age estimates in general and for parameters affecting rates of morphological and molecular evolution (table 1 and electronic supplementary material, tables S1, S2). Making the informative priors penalize ghost lineages even more than shown here (for instance, by changing the prior on *f* from Beta(100,1) to Beta(10 000,1)) did not change parameter estimates, including divergence time estimates, appreciably.

### (e) Fossil contribution to dating

**Figure 6.**
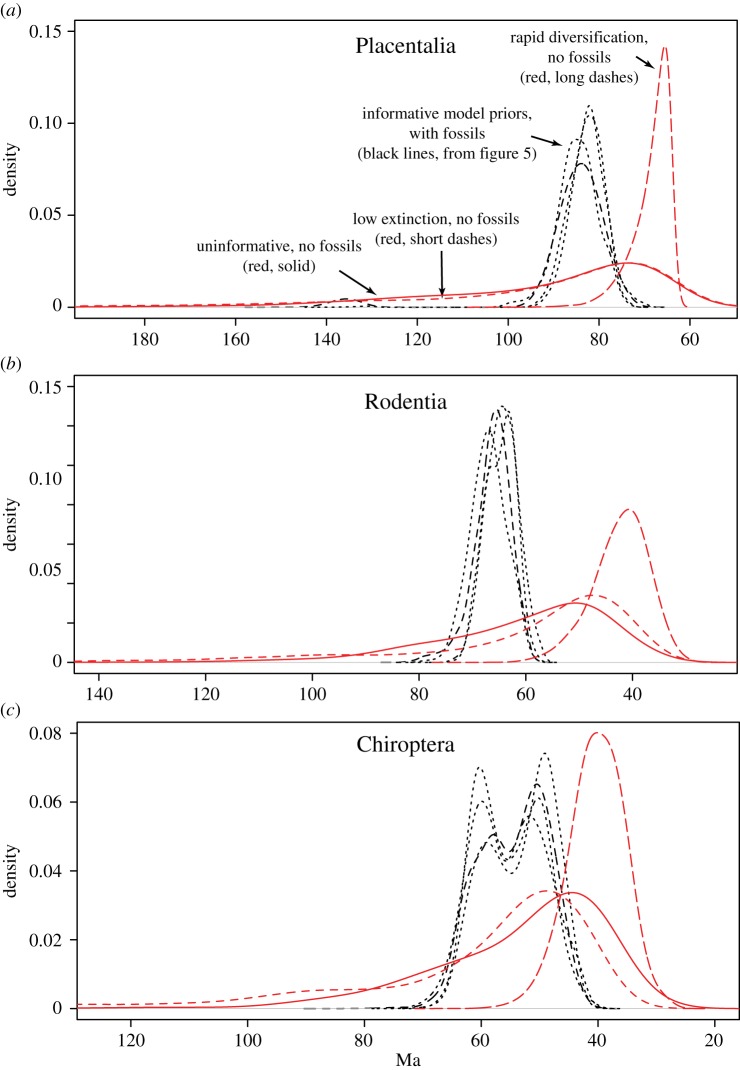
The influence of fossils on divergence time estimates. (*a*) Divergence time estimates (posterior densities) for crown Placentalia in analyses with fossils (black lines) and without fossils (red lines), and under different priors on diversification and fossil sampling parameters. Scenarios with fossils correspond to those in figure 5, except that the uninformative-prior scenario is omitted for clarity. Scenarios with fossils are equivalent, when applicable, except that fossil-related parameters are excluded. (*b*) Ditto for crown Rodentia. (*c*) Ditto for crown Chiroptera. Despite the conflict between morphology and molecules, making it challenging to place the fossils correctly in the extant tree, the fossils nevertheless contribute important dating information. Specifically, they tend to make divergence time estimates more robust to variations in prior assumptions and more precise (black lines) than if dating is solely based on information from extant taxa (red lines). (Online version in colour.)

To assess the fossil contribution to dating, we compared divergence time estimates obtained with and without fossils included, using identical priors (except for the fossil sampling prior required by the FBD model, which is inapplicable when fossils are excluded). The results show that fossils make divergence time estimates more precise and more robust to variations in prior assumptions, given that DRA is addressed properly (figure 6).

Without fossils, a vague prior or a low-extinction prior both give diffuse posterior distributions that do not contain much dating information, whereas a rapid-diversification prior pulls the dates so strongly towards the recent that the tree gets inappropriately compressed against the minimum root age constraint (which was set to 64 Ma; see the inferred age of Placentalia, figure 6*a*). The result is divergence time estimates that are quite precise but probably too young given the fossil record. For instance, without fossils, the rapid diversification prior suggests an age of 42 (33, 53) Ma for crown-group rodents (figure 6*b*) while even conservative inference from the fossil record suggests at least 52.5 Ma (based on several ‘primitive’ hystricognaths [10]). Furthermore, the same prior places bats at 40 (31, 50) Ma (figure 6*c*), whereas the fossil record indicates a minimum of 45 Ma based on the age of *Tanzanycteris* or possibly 47 Ma based on the slightly older *Tachypteron*. When fossils are included in the analysis, the divergence times arguably agree better with the fossil record; at least, they meet the expected minimum age requirements for both rodents and bats (figure 6*b*,*c*).

### (f) Effect of conflict between morphology and molecules

To tease apart the DRA effect caused by a vague FBD prior and the part caused by the difficulty of reconciling the morphological data and fossil ages with the extant tree, we fixed the tree topology using the ending tree from one of the informative FBD prior analyses (the rapid diversification prior), and then inferred the divergence times with morphological data excluded. When topology was unconstrained (except for the rooting constraint) and morphology was included, vague priors placed the placental origin at approximately 118 Ma, whereas informative priors placed the origin at around 85 Ma (figures 5*a* and 6*a*). The topologically constrained analysis under the vague FBD prior and without morphological data placed the origin at around 88 Ma. Thus, the main driver of DRA in this dataset appears to be the difficulty of fitting the required amount of morphological evolution under the Mk model onto what would otherwise appear to be the most reasonable clock tree.

### (g) Insights from alternative models

Unlinking morphological and molecular-relaxed clocks had no major effect on the inferred divergence times, indicating that it is problems with the morphological model itself that drive DRA, and not inappropriate linking of morphological and molecular rate variation. The skyline FBD model with vague priors was not able to pick up a signal of rapid placental radiation around the K–Pg event. Instead, the skyline FBD model actually made the DRA effect worse, presumably by adding more independent parameters with vague priors.

### (h) Divergence time estimates

**Figure 7.**
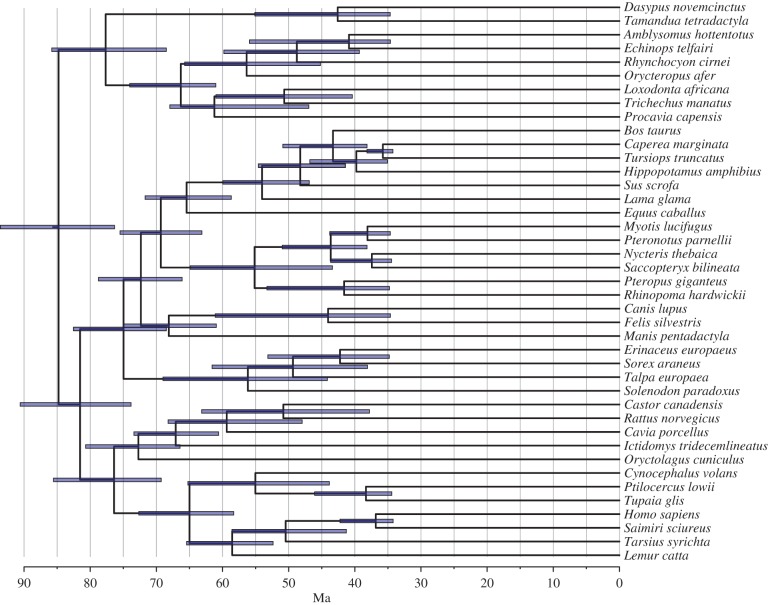
Divergence time estimates for the placental radiation under TED. Results are shown for the rapid diversification model, but results are similar for other informative priors on the diversification process (figures 5 and 6). The estimated age for placentals (crown eutherians) is around 86 Ma, younger than in most molecular-clock analyses but still a considerable distance from the post-K–Pg-boundary estimate favoured by some palaeontologists. Note that the crown groups of most placental orders postdate the K–Pg boundary, consistent with the fossil record. (Online version in colour.)

Overall, the total-evidence analysis (under informative priors) provides divergence time estimates that are younger than those obtained under most molecular-clock analyses published to date, even though the inferred ages are still older than what is obtained from analyses that minimize ghost lineages, especially for the deepest splits in the tree (figure 7 and table 1). Some shallow divergences have young means, but the highest posterior density almost always overlaps the fossil record expectations. No placental orders are inferred to predate the K–Pg boundary except for Rodentia, which barely reaches into the Cretaceous. In total, 12 extant placental lineages are inferred to cross the K–Pg boundary but only eight of them pass beyond 70 Ma.

The placental crown radiation is dated to 85 (76, 93) Ma under the rapid diversification prior, and the other informative total-evidence priors give very similar results (figure 6*a* and electronic supplementary material, table S1). Because the same rapid diversification prior places the radiation of placental mammals at 67 (64, 77) Ma when fossils are excluded from the analysis (figure 6*a*), it is clear that the fossil data provide considerable resistance against a scenario where the entire placental radiation took place in the Palaeogene.

### (i) Positions of fossils

**Figure 8.**
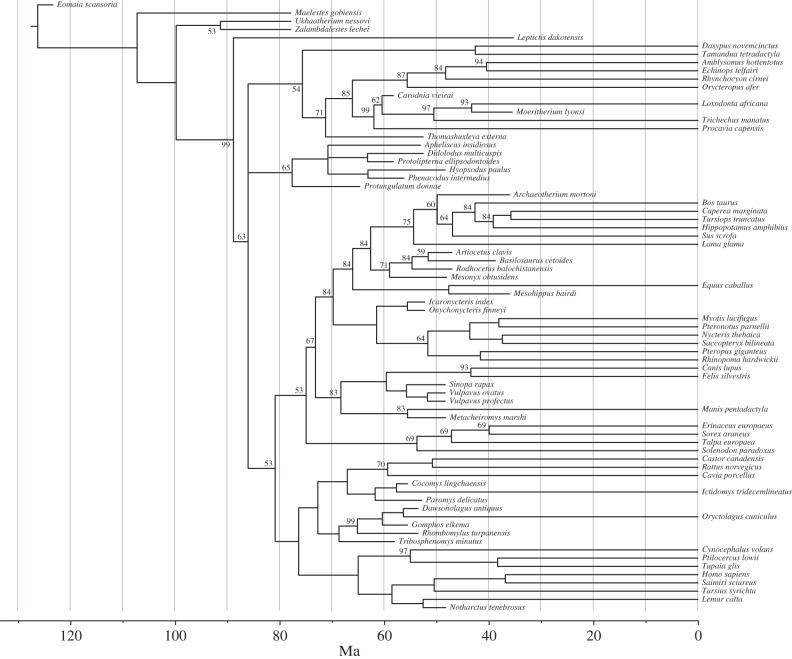
Inferred phylogenetic affinities of fossils. Despite the plasticity in the morphological data and/or the imperfection of our models of morphological evolution, the placements of fossils suggested by total-evidence analyses largely agree with the views expressed by palaeontologists (see text for more detailed discussion). Results are shown for the model assuming rapid diversification, but fossil placements were similar for other informative priors on the diversification and fossil sampling processes.

Under a total-evidence analysis, some fossils float around in the tree while the rich morphological data succeed in placing many others with considerable certainty (figure 8). Most of the fossil placements are quite consistent with palaeontological interpretations of the fossil record. The more notable exceptions include a monophyletic grouping of ‘condylarths’ usually thought of as paraphyletically or polyphyletically distributed in the tree, from presumed basal or stem placentals (*Protungulatum*) to South American (*Didolodus*, *Protolipterna*) and Northern Hemisphere (*Hyopsodus*, *Phenacodus*, *Apheliscus*) relatives of afrotherian or laurasiatherian ungulates. Another pair of presumed ungulate fossils (*Thomashuxleya*, *Carodnia*) are attracted towards basal Afrotheria lineages (the same effect is seen in O'Leary *et al*. [8]). Furthermore, three fossils that are usually considered to be crown or stem cetaceans [30], *Rhodocetus*, *Artiocetus* and *Basilosaurus*, are pulled deeper down into the tree in our analysis, forming a group of stem artiodactyls together with *Mesonyx*, a traditional mesonychid sister taxon to cetaceans. Most other fossil placements would seem to be in line with expectations. *Leptictis*, for example, is sister to crown placentals, which is more consistent with the traditional view that its closest affinities are with Late Cretaceous eutherians, such as *Gypsonictos* [31]. However, it seems that the large number of cranial and dental characters in the dataset might have resulted in the grouping of *Mesonyx* with fossil cetaceans, which is contradicted by major basicranial and ankle character complexes.

## 4. Discussion

### (a) Reconciling phenomics and genomics

TED is based on simultaneous treatment of all available evidence—including morphology (phenomics), molecules (genomics) and the fossil record—in an integrated statistical analysis [2–4]. The strong conflict between morphological and molecular signal in mammals is clearly problematic in such a context, but at least a unified statistical framework provides the platform needed to understand the nature of the different signals and to make progress in reconciling them.

Springer *et al*. [13] highlighted the fact that the impressively large phenomic dataset assembled by O'Leary *et al*. [8]—when analysed using parsimony methods ([8]: electronic supplementary material, figure S2; [13]: figure 1)—supports a number of groups that many workers today interpret as reflecting convergent ecological adaptations rather than as genealogical relationships. The problematic assemblages include an ‘insectivore’ group (where most workers would probably agree that the small Madagascar hedgehog is misplaced), an ‘ant and termite eating’ group (pulling together unrelated forms like the pangolin, aardvark, anteater and armadillo), a ‘tree-dwelling group’ (placing bats with primates and their relatives) and an ‘ungulate’ group (including taxa such as elephants, sirenians and the rock hyrax alongside artiodactyls and perissodactyls) [13].

The results from our statistical reanalysis of the phenomic data (figure 1*c*) differ in minor details from the parsimony analysis, but confirm that the phylogenetic signal is weak and apparently influenced by convergent adaptation to similar life histories. Adding fossils to the statistical analysis (electronic supplementary material, figure S2*a*) helps improve some parts of the morphology tree, but the tree still contains most of the unexpected groupings found in the parsimony analysis and in the statistical analysis of extant taxa.

Adding molecular data to the statistical analysis appears to resolve most of the phylogenetic artefacts caused by morphological convergence (figures 1*a* and 8; electronic supplementary material, figure S2*b*). Nevertheless, as long as there is significant mismatch between the results of statistical phylogenetic analysis of the morphological data and the expert evaluation of the same data by comparative anatomists and palaeontologists, we should be concerned that our stochastic models of morphological evolution might be inadequate. Indeed, it is easy to see a number of serious potential shortcomings in the standard Mk model for the evolution of discrete morphological characters, which we used here.

First, the Mk model does not accommodate directional evolution, which is readily shown to occur in some morphological datasets over the timescales involved here [32]. Perhaps more importantly, the variation in the rate of morphological evolution across characters and lineages is poorly understood and has probably been inadequately modelled so far. For instance, we explored models assuming that morphological and molecular clocks are either the same or completely independent. In reality, however, the morphological and molecular rates are likely to be neither identical nor independent but correlated. Furthermore, we used an uncorrelated relaxed clock model even though there is much to suggest that a significant component of rate variation across lineages is constrained by long-term autocorrelation, and failure to accommodate for this is likely to bias inferred divergence times. Separately, we explore more sophisticated models of rate variation that account for both long-term and short-term rate variation and their effects on dating the placental radiation [33]. Analyses under such models suggest that the naive uncorrelated model used in this paper does not fully recognize the rapid evolutionary rate of rodents, presumably owing to their small body size and therefore short generation time, which is strongly phylogenetically constrained. The effect is that the current analysis probably overestimates the age of crown rodents, so that the conclusion that they predate the K–Pg event may be erroneous [34].

Another significant source of error in current stochastic models of morphological evolution is the assumption that characters evolve independently of each other. In reality, morphological evolution is likely to be strongly constrained by various types of character dependencies. When single evolutionary events can affect large groups of correlated characters, the probability of unrelated lineages evolving similar morphologies is much higher than if all characters evolve independently, and failure to account for this is likely to lead to errors in phylogenetic inference akin to the effect of long-branch attraction. The symptoms may include erroneous or overconfident topological inference, as well as excessively precise and biased branch length estimates, both of which may be problematic in TED. The grouping of ecologically similar but unrelated forms in the phenomic tree (figure 1*c*), and the fact that some fossil placements seem to put undue weight on highly correlated dental features (figure 8), could both be explained as artefacts in our analyses caused by an inappropriate assumption of evolutionary independence among morphological characters.

Accounting for interdependence among morphological characters is challenging, but such work will definitely be worthwhile given the importance of realistic models of morphological evolution for accurate dating of phylogenies with fossils. Denser taxon sampling, especially of fossils, might help to some extent but to really solve the problem we need to get at the correlation structure itself, either by inferring it or by modelling it in the prior. Methods for learning the correlation structure of small numbers of discrete (morphological) characters are well known [35], and there has been considerable progress in sampling from models accounting for large sets of discrete (molecular) characters with known correlation structure [36,37]. The challenge is to find computationally feasible ways of accommodating large numbers of morphological characters with unknown correlation structure, or to find reasonable *a priori* representations of the way in which morphological characters are likely to coevolve.

### (b) Deep root attraction

Our results suggest that there is, indeed, a tendency for TED to produce unrealistically old divergence time estimates under certain conditions, the effect we term DRA. It appears that DRA is caused by the combination of vague priors with various types of model inadequacies. Specifically, it seems that the strong conflict between morphological and molecular signals is one of the primary drivers of DRA in the data of O'Leary *et al*. The conflict appears to be caused to a large extent by a failure to account for the evolutionary dependencies among morphological characters, resulting in positively misleading signal due to functional convergence when morphology is analysed alone. Even though most of the topological artefacts are corrected when the morphological data are combined with molecular data, there is a large amount of unlikely convergent evolution in morphology to explain in the combined-data tree. This results in a push towards older dates, giving more evolutionary time for unlikely events to occur. If this interpretation is correct, then we would expect DRA to be more severe the larger the morphological dataset, with massive phenomic datasets like the one analysed here representing the worst case as they are likely to include large numbers of correlated characters.

It is obvious from our results that a failure to account for diversified sampling of extant taxa can also cause severe DRA in the dating of higher-level phylogenies. This is consistent with results for the dating of the early radiation of Hymenoptera (ants, wasps and bees) [5] and an earlier analysis of the placental radiation [15]. Thus, accounting for tip sampling biases is important in addressing DRA. The smaller the fraction of taxa sampled for the analysis, the more important it probably becomes to model the sampling biases correctly. In contrast, making the TED model more realistic by addressing speciation, extinction and fossil sampling using the FBD process is not sufficient to remove the effects of DRA, at least not under uninformative priors. When the possibility of different (elevated) diversification rates just after the K–Pg event is accommodated using the skyline FBD model, DRA becomes even stronger in our analyses, presumably because of the addition of more parameters with vague priors. Thus, it appears that the signal in the data concerning diversification processes is not strong enough to overcome DRA under standard FBD priors. Similarly, uncoupling the morphological and molecular clocks has little effect on DRA.

Fixing the placement of fossils based on palaeontological expertise and not using the morphological data in divergence time estimation appears to be one way to address DRA. Doing so, one would still rely on a principled approach in integrating information on fossil ages, molecular data and diversification processes in the analysis [33,38]. However, ultimately, the purpose of TED is to explicitly formalize all aspects of the macroevolutionary process, including the evolution of morphological characters, and to use this information in integrating over the uncertainty in the placement of fossils.

While waiting for better models of morphological evolution, our analyses show that a simple way to control DRA is to increase the penalty for unobserved ghost lineages in order to correct for model inadequacies and to augment weak signal in the data. The exact method for penalizing ghost lineages appears to be unimportant; the end result is very similar with respect to the posterior probability distribution. Increasing the penalty beyond the point where a phase shift occurs in the posterior appears to have no effect; thus, it is certainly not possible to get whichever divergence time estimates you like by these types of prior modifications.

Of the informative priors we explored for the placental data, presumably the assumption of rapid net diversification up to the last sampled speciation event is the one that comes closest to a reasonable prior model assumption. However, we do not argue that any of the informative priors we used here is necessarily a reasonable model prior for the placental data; the penalizing effect on ghost lineages is the important factor, which can be used as an *ad hoc* fix for DRA when the primary interest is divergence time estimation.

Clearly, the informative priors are partly compensating for the fact that we are not accounting for all of the relevant background information in the rest of the model. Consider, for instance, that the strongest evidence we have for an exclusively or largely Palaeogene radiation of placentals is the observation that we have found so many placental fossils in the Palaeogene but none in the Cretaceous (at least not definitive crown placentals). The ratio of the number of fossils found before and after the critical time period is one of the critical factors determining the odds against a placental origin in the Cretaceous. In the analysis, we discuss in this paper, the magnitude of this factor is estimated from the distribution in time of a sample of some 30 fossils, a miniscule subset of the placental species actually present in the fossil record. Clearly, the odds against a Cretaceous origin of placentals will be grossly underestimated using such a small fossil sample. We should really correct for this by using a more informative prior (representing more of the background information) or by including more fossils in the analysis.

There are several other ways of making the FBD prior more informative. One interesting possibility is to consider models that would accommodate spatially structured fossil sampling probabilities. This would allow for ghost lineages to be penalized less when fossil records are expected to be poor, such as for the afrotherian stem; there are no known mammal fossils from the Late Cretaceous of Africa. A fairly obvious improvement would be to consider diversification models allowing diversification rates to vary over time, and extend the sampling of extant and extinct taxa to increase our ability to infer such patterns.

It remains an open question whether a more powerful empirical signal concerning the diversification and sampling processes is enough to compensate for the DRA effect caused by other model inadequacies, such as the failure to account for non-independence in morphological evolution. However, it is clear that we can learn much from studying all these model aspects in an integrative context, such as that of TED.

Although we have pointed to several reasons to expect DRA in TED, there are also TED effects that counteract DRA. In particular, when morphological evolution can be modelled accurately, fossil morphology is well known, and fossils are close to predicted ancestral phenotypes, fossils will tend to pull speciation events towards the recent. Thus, although it is quite possible that DRA is a common problem in TED [6], each case is potentially unique and warrants a fresh look before any definite conclusions are drawn.

### (c) Dating the placental radiation

We find it encouraging that the total-evidence analysis presented here produces divergence time estimates that match the fossil record closely despite the fact that we did not use any direct constraints on node dates. The explosive radiation scenario favoured by O'Leary *et al*. [8,14] places the placental origin just after the K–Pg mass extinction at 66 Ma. It requires both net diversification rates and rates of molecular evolution during the early phases of placental evolution that are unlikely considering other sources of evidence on mammalian evolution [13]. O'Leary *et al*. contrasts the explosive radiation scenario with a much-cited molecular-clock study that uses node dating and places the origin of crown placentals at 101 Ma [9]. Our TED analysis, under informative priors, halves the gap between these two estimates, from approximately 36 to 16–19 Myr (table 1 and electronic supplementary material, table S1). Our estimates for the timing of the subsequent Palaeogene radiation of placentals come even closer to the dates inferred from the fossil record according to O'Leary *et al*. [8]. Our estimate for the timing of the primary interordinal radiation of placental mammals that Meredith *et al*. [9] date at approximately 83 Ma, falls close to the 66 Ma K–Pg boundary, with 95% highest posterior densities of several major clades—including Euarchonta, Eulipotyphla, Afrotheria, Euungulata (artiodactyls, perissodactyls) and Ferae (carnivorans, pangolins)—overlapping this boundary.

We note that our divergence time estimates agree fairly well with a recent phylogenomic study using a rich set of ‘soft’ node calibrations [10], but our estimates tend to have more uncertainty associated with them. The increased uncertainty is not due to the fact that we used a smaller molecular dataset than dos Reis *et al*. [10]. In our analyses, effective branch lengths (measured in expected substitutions per site or character) in the extant tree were estimated with high precision (e.g. electronic supplementary material, figure S1*c*) and were almost invariant across models. Thus, almost all of the dating uncertainty comes from other parts of the TED model. So are the estimates from our analyses more or less reliable than those from the node dating study based on phylogenomic data?

This is not an easy question to answer. Although TED is an elegant and theoretically satisfying approach, TED is not necessarily better than node dating. It should be possible to summarize the information in the fossil record quite accurately, particularly in a set of soft node calibrations as used in dos Reis *et al*. [10]. However, deriving these calibrations is a difficult exercise that is prone to error and various biases, and that could possibly result in exaggerated precision. Thus, it is conceivable at least that the general uncertainty levels in our analyses are more realistic, despite the modelling problems that we encountered.

Regardless of this particular comparison, it is clear that the general advantage of the TED approach is that it offers a unified statistical framework for understanding the relative contributions of different sources of evidence, and for combining them using the common arbiter of probability. This should make it easier to identify modelling problems, to improve the reconciliation among conflicting data signals, to successively account for more of the available evidence and ultimately to improve the accuracy of divergence time estimation.

## Supplementary Material

Supplementary figures and tables

## Supplementary Material

Appendix S1

## Data accessibility

The datasets and MrBayes scripts used in this paper were uploaded as part of the electronic supplementary material.
