# Supplementary material for: Closing the gap between rocks and clocks using total-evidence dating
Source: Philos Trans R Soc Lond B Biol Sci. 2016 Jul 19;371(1699):20150136. doi: 10.1098/rstb.2015.0136 (PMC4920337; doi:10.1098/rstb.2015.0136)
Supplement: Supplementary figures and tables [file rstb20150136supp1.pdf]

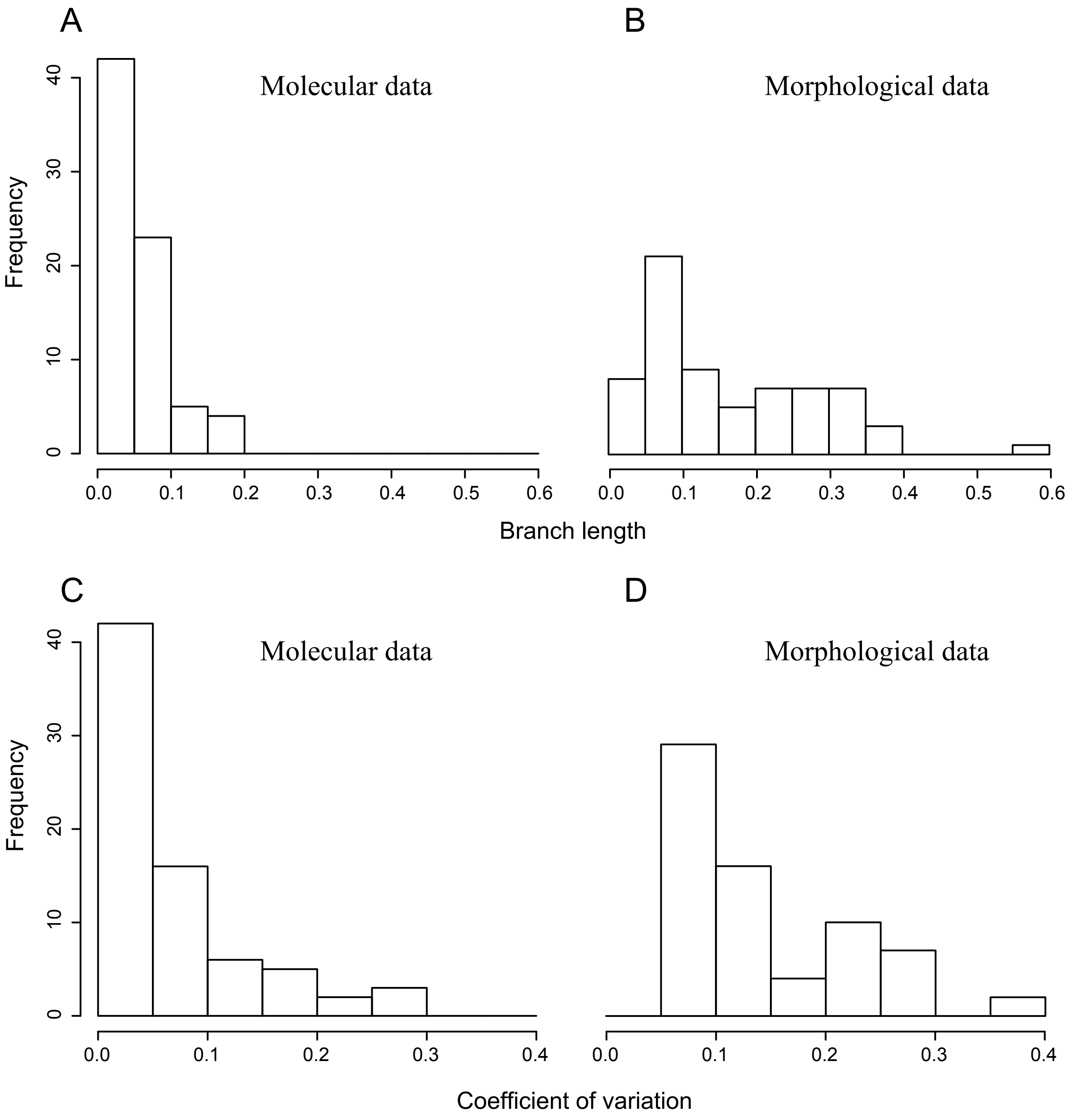

Figure S1. **Estimated branch lengths in non-clock analyses of extant taxa**. (A) Estimated mean branch lengths in analysis of molecular data. (B) Ditto in analysis of morphological data. (C) Estimated coefficients of variation for branch lengths in analysis of molecular data. (D) Ditto in analysis of morphological data. Only branches present in more than 95 % of sampled trees shown (n = 72 for molecular analysis, n = 68 for morphological analysis). Note that morphological branch lengths are considerably longer than molecular branch lengths (A versus B), but still quite precise (C versus D).

Ronquist, F *et al.* 2016. Closing the gap between rocks and clocks using total-evidence dating. *Phil. Trans. R. Soc. B* doi: 10.1098/rstb.2015.0136

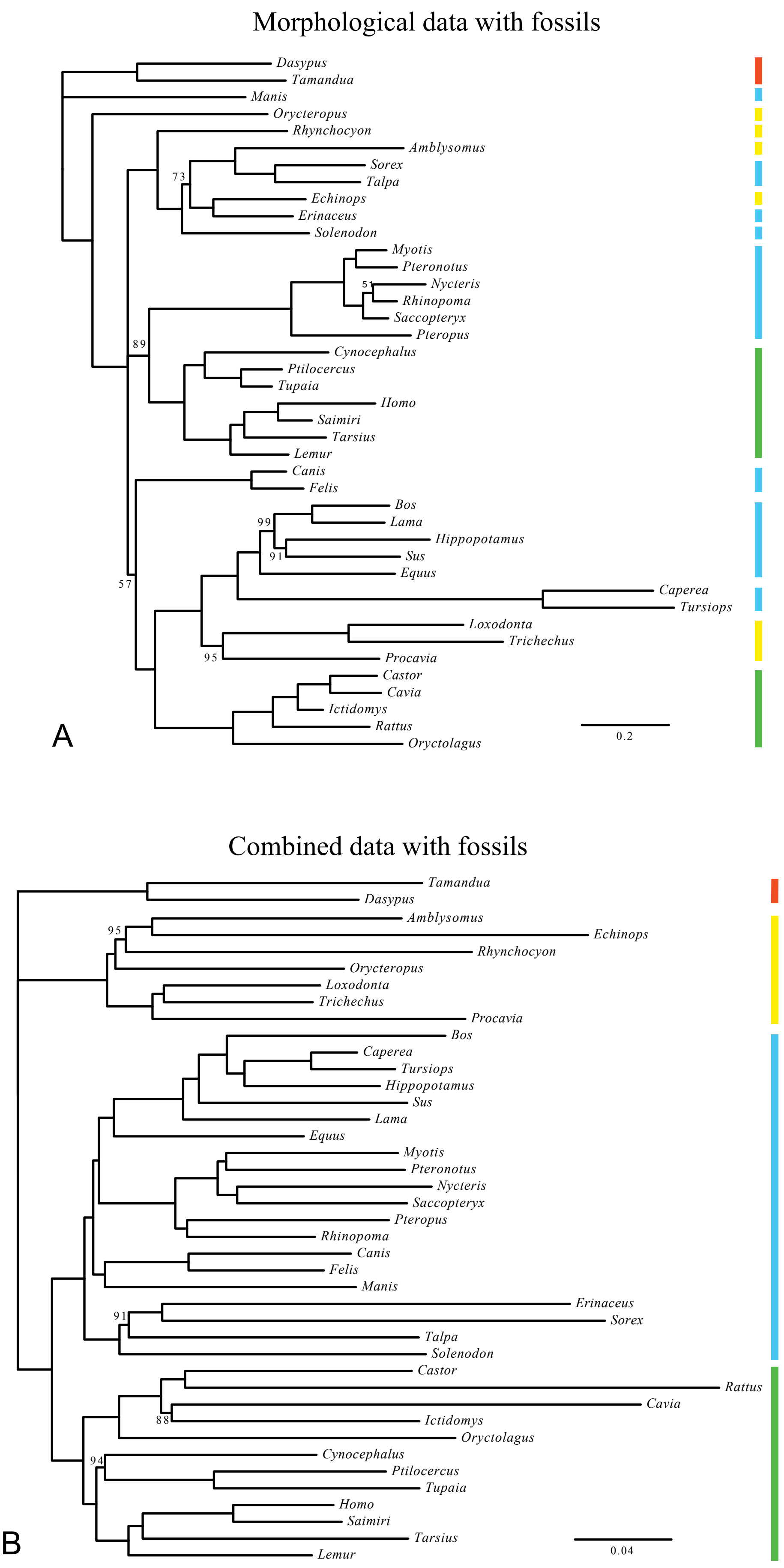

Figure S2. **Phylogenetic relationships among extant taxa in non-clock analyses including fossil taxa**. (A) Analysis based on morphological characters only. (B) Analysis based on a combination of morphological and molecular data.

Table S1. **Prior and posterior distributions for the ages of key clades under three informative prior models (low extinction, high fossilization, or low extinction and high fossilization combined)**. The induced prior distributions were estimated from the full model, with fossils included, using Markov chain Monte Carlo sampling with the log likelihood of observed characters set to 0.0. Estimated distributions are summarized by the median followed by the 95 % region of highest posterior density.

	Low extinction		High fossilization		Low extinction, high fossilization	
**Clade**	**Induced prior**	**Posterior**	**Induced prior**	**Posterior**	**Induced prior**	**Posterior**
Placentalia	81 (58, 96)	84 (73, 94)	81 (56, 98)	83 (76, 90)	83 (61, 126)	82 (74, 90)
Xenarthra	38 (33, 53)	42 (35, 54)	38 (33, 52)	41 (35, 52)	38 (34, 53)	42 (35, 54)
Afrotheria	47 (37, 62)	66 (60, 72)	46 (37, 60)	65 (60, 72)	47 (38, 61)	64 (60, 71)
Afroinsectiphila	40 (35, 50)	56 (46, 66)	40 (35, 50)	55 (45, 65)	40 (35, 50)	53 (45, 64)
Paenungulata	39 (34, 49)	62 (49, 69)	39 (34, 49)	60 (47, 67)	40 (35, 50)	57 (46, 66)
Boreoeutheria	61 (51, 81)	81 (71, 91)	60 (50, 82)	80 (74, 86)	63 (52, 84)	79 (72, 87)
Laurasiatheria	55 (44, 65)	74 (66, 86)	55 (44, 65)	74 (69, 81)	56 (46, 68)	73 (65, 80)
Eulipotyphla	41 (35, 51)	57 (41, 72)	41 (35, 49)	55 (44, 66)	41 (35, 50)	55 (43, 68)
Euungulata	43 (37, 53)	65 (59, 75)	43 (37, 53)	64 (58, 69)	44 (37, 54)	63 (58, 68)
Cetartiodactyla	40 (36, 48)	54 (47, 61)	40 (36, 47)	51 (44, 58)	41 (36, 49)	50 (44, 57)
Chiroptera	42 (36, 51)	54 (45, 65)	42 (37, 52)	55 (45, 64)	43 (37, 52)	52 (44, 63)
Ferae	39 (34, 49)	67 (60, 79)	39 (34, 48)	66 (60, 72)	40 (35, 49)	65 (58, 71)
Carnivora	36 (33, 42)	42 (35, 61)	36 (33, 41)	40 (35, 52)	36 (34, 41)	40 (35, 50)
Euarchontoglires	52 (41, 63)	76 (68, 84)	51 (41, 62)	74 (69, 80)	53 (41, 64)	74 (68, 79)
Glires	43 (36, 54)	72 (66, 80)	43 (36, 52)	71 (66, 76)	43 (36, 54)	71 (65, 76)
Rodentia	39 (35, 46)	66 (60, 73)	39 (35, 45)	65 (60, 70)	39 (35, 47)	64 (59, 71)
Euarchonta	44 (37, 55)	65 (55, 73)	44 (37, 54)	61 (54, 70)	45 (38, 56)	63 (56, 70)
Primates	39 (34, 46)	57 (51, 65)	39 (35, 46)	55 (50, 61)	39 (35, 46)	56 (51, 63)

Table S2. **Prior and posterior densities for rate parameters under five TED model priors**. The prior density is given as the median and the 95 % credible interval of the distribution associated with each parameter. Estimated posterior distributions are summarized by the median value followed by the 95 % region of highest posterior density. For the rate multipliers, we used a flat Dirichlet prior on the rate times the proportion of the characters in that partition (as this quantity must sum to 1.0 across partitions). This means that the partitions with a smaller number of characters (untranslated, morphology) have a higher variance and therefore a higher median in the prior than the partitions with a high number of characters (codon positions 1–3).

		**Estimated posterior under different TED model priors**				
**Parameter**	**Prior**	**Uninformative**	**Low extinction**	**High fossilization**	**Low extinction, high fossil.**	**Rapid diversification**
Clock rate (per site per Gyr)	2.48 (0.93, 6.60)	1.54 (1.06, 1.94)	2.35 (2.03, 2.65)	2.42 (2.14, 2.72)	2.43 (2.15, 2.79)	2.27 (2.05, 2.53)
Rate multiplier, untranslated	2.40 (0.10, 9.10)	0.56 (0.53, 0.59)	0.56 (0.53, 0.59)	0.56 (0.53, 0.59)	0.56 (0.53, 0.59)	0.56 (0.53, 0.59)
Rate multiplier, codon pos. 1	0.58 (0.02, 2.19)	0.59 (0.57, 0.60)	0.59 (0.57, 0.60)	0.59 (0.57, 0.60)	0.59 (0.57, 0.60)	0.59 (0.57, 0.60)
Rate multiplier, codon pos. 2	0.58 (0.02, 2.19)	0.52 (0.50, 0.53)	0.52 (0.50, 0.53)	0.52 (0.50, 0.53)	0.52 (0.50, 0.53)	0.52 (0.50, 0.53)
Rate multiplier, codon pos. 3	0.58 (0.02, 2.19)	1.32 (1.30, 1.34)	1.32 (1.30, 1.34)	1.32 (1.30, 1.34)	1.32 (1.30, 1.34)	1.32 (1.30, 1.34)
Rate multiplier, morphology	1.45 (0.06, 5.49)	2.93 (2.86, 2.93)	2.93 (2.86, 3.01)	2.94 (2.86, 3.01)	2.94 (2.86, 3.01)	2.93 (2.86, 3.01)
Variance of relaxed clock (x 1000)	69.3 (2.5, 368.9)	11.5 (8.0, 15.7)	18.8 (13.7, 25.2)	20.0 (14.8, 26.1)	20.5 (15.3, 26.6)	18.0 (13.6, 23.1)
